# Protection of pigs against *Taenia solium* cysticercosis by immunization with novel recombinant antigens

**DOI:** 10.1016/j.vaccine.2012.04.019

**Published:** 2012-06-06

**Authors:** Charles G. Gauci, César M. Jayashi, Armando E. Gonzalez, Julia Lackenby, Marshall W. Lightowlers

**Affiliations:** a University of Melbourne, Faculty of Veterinary Science, Werribee, Victoria 3030, Australia; b School of Veterinary Medicine, Universidad Nacional Mayor de San Marcos, Lima, Peru

**Keywords:** ELISA, enzyme-linked immunosorbent assay, FnIII, fibronectin type III, GST, glutathione S-transferase, MBP, maltose binding protein, Recombinant, Vaccine, Antigen, Parasite, *Taenia*, Cysticercosis

## Abstract

Recombinant antigens from the oncosphere stage of the parasite *Taenia solium* were expressed in *Escherichia coli*. The TSOL16, TSOL45-1A and TSOL45-1B recombinant antigens, each consisting of fibronectin type III (FnIII) domain S, were produced as fusion proteins with glutathione S-transferase (GST) and maltose binding protein (MBP). Groups of pigs were immunized twice with the GST fusions of the antigens and boosted a third time with the MBP fusions prior to receiving a challenge infection with *T. solium* eggs. The TSOL16 antigen was found to be capable of inducing high levels of immunity in pigs against a challenge infection with *T. solium*. Immunological investigations identified differences in immune responses in the pigs vaccinated with the various antigens. The results demonstrate that the TSOL16 antigen could be a valuable adjunct to current porcine vaccination approaches and may allow the further development of new vaccination strategies against *T. solium* cysticercosis.

## Highlights

► Experimental *Taenia solium* challenge of piglets vaccinated with recombinant antigens. ► Protection of piglets immunized with the TSOL16 recombinant antigen. ► Assessment of antibody responses in pigs vaccinated with recombinant antigens.

## 1 Introduction

Cysticercosis in humans occurs following infection with the cestode parasite *Taenia solium* and is a major cause of neurological disease worldwide [1]. It is associated with poor living standards and poor sanitation, occurring in developing countries where free-roaming pigs and the lack of latrines contribute to transmission of the parasite from pigs to humans. Vaccination of pigs has been proposed as a potential tool to control transmission of *T. solium* from pigs to humans, in order to reduce the incidence of human neurocysticercosis [2,3]. A recombinant subunit vaccine, the TSOL18 antigen, has been shown to be highly effective in preventing infection of pigs in controlled experimental trials [4,5]. The TSOL18 vaccine is also highly effective as a porcine vaccine against naturally acquired infection with *T. solium* [6].

Other recombinant antigens have also been cloned from the larval oncosphere stage of the *T. solium* parasite. These include a family of related antigens, designated TSOL45, that have been identified as protein isoforms, some of which result from alternatively spliced mRNA transcripts in the oncosphere [7]. Analyses of the TSOL45 mRNAs have identified a variety of oncosphere proteins encoding two, one or no fibronectin type III (FnIII) domains. One of these gene products, TSOL45-1A, that is not alternatively spliced and contains two FnIII domains, has been shown to protect pigs against experimental infection with *T. solium* [4,5]. Other antigens encoded by the TSOL45 gene family have not yet been evaluated for their ability to protect pigs against infection with the *T. solium* parasite.

The TSOL16 antigen is a third *T. solium* antigen type that has been cloned from oncospheres and the encoding gene has been characterized [8]. It was isolated from *T. solium* following demonstration of the ability of a homologous recombinant antigen, To16, to confer protection of vaccinated sheep against a related parasite, *Taenia ovis* [9]. TSOL16 appears to be specifically expressed in the oncosphere life cycle stage of *T. solium* [10] and is associated with penetration gland cells [11].

Although the development of a porcine vaccine based upon the TSOL18 antigen is at an advanced stage, nevertheless it remains important to evaluate the potential for other antigens to protect pigs against *T. solium*. For example, widespread application of a vaccine based on a single immunogen could potentially select for genetic variants of *T. solium* having reduced susceptibility to the vaccine. Application of a vaccine incorporating multiple, antigenically unrelated immunogens would be expected to reduce the likelihood of selection of resistant parasites, in a manner analogous to the use of different anthelmintics to reduce selection for resistance [12]. Currently available evidence [13] does not suggest that genetic variability in the TSOL18 protein would be a problem during the initial application of the TSOL18 vaccine, however evaluating the ability of other recombinant proteins to complement TSOL18 would add to the potential reliability of vaccination as a control measure for *T. solium*.

The aims of this study were to evaluate whether the TSOL16 protein could be used to protect pigs against infection with *T. solium* and to determine whether a protein related to the TSOL45-1A antigen and encoded by a splice variant lacking one of two FnIII domains (TSOL45-1B) retains the ability to protect pigs against cysticercosis.

## 2 Materials and methods

### 2.1 Preparation of recombinant antigens

The TSOL16 cDNA was originally cloned from *T. solium* oncosphere mRNA as described in [8]. Two related TSOL16 cDNAs were first isolated, designated TSOL16A and TSOL16B, which differed at two positions in their predicted amino acid sequences [8]. The TSOL16A cDNA was selected for expression in *Escherichia coli* since the substituted amino acids were identical in sequence to To16 from *T. ovis*, a related antigen that has been previously shown to be host protective in sheep [9]. The encoded TSOL16A protein contains hydrophobic amino acids within a predicted secretory signal at the N-terminus. In order to enable efficient expression of the TSOL16A protein in *E. coli*, PCR amplification was used to produce a cDNA construct encoding a modified form of the antigen that lacked the 16 N-terminal amino acids of the secretory signal. The procedure that was followed is similar to that outlined in [14] and utilized the following PCR primers: 5′CCG GAA TTC GAT GGA TTC GGT GAA TTT GGC G3′; 5′CCG CTC GAG CAT GCA ATG GAA TCC CAG AAG3′. This truncated TSOL16A cDNA (herein referred to as TSOL16 with respect to the cDNA and encoded protein) was cloned directionally into the *Eco*RI and *Xho*I sites of pGEX-1TEX and transformed into *E. coli* JM109 strain by electroporation. Use of the pGEX plasmid allowed expression and purification of TSOL16 as a fusion with glutathione *S*-transferase (GST) [15].

The truncated TSOL16 cDNA was excised from pGEX-1 by digestion with *Eco*RI and *Xho*I, and cloned into *Eco*RI/*Sal*I-digested pMAL-C2. The pMAL-C2 plasmid allowed expression and purification of TSOL16 as a fusion with maltose binding protein (MBP) [16]. The plasmid construct was transformed into *E. coli* JM109.

The TSOL45-1A protein was cloned into the pGEX and pMAL-C2 plasmids, and expressed in *E. coli* as a fusion protein with GST and MBP as described in [4]. The TSOL45-1A fusion proteins lacked 16 N-terminal amino acids that encoded a predicted secretory signal.

The TSOL45-1B cDNA was originally cloned from *T. solium* oncosphere mRNA as described in [7]. TSOL45-1B lacked exon II of the *TSOL45-1* gene. PCR amplification was used to produce a cDNA construct that encoded a protein also lacking the 16 N-terminal amino acids of the secretory signal. The following PCR primers were used to amplify TSOL45-1B for cloning into pGEX and pMAL as described above: 5′CCG GAA TTC GGA AAC CAC AAG GCA ACA TC3′; 5′CCG CTC GAG GGA AAT GGG CAT TGA CCG3′.

*E. coli* cultures expressing TSOL16, TSOL45-1A and TSOL45-1B were prepared and recombinant fusion proteins were purified as detailed in [14].

Freeze-dried aliquots of antigens were prepared by the addition of Quil A adjuvant (1 mg per dose) and a sixfold (w/w) amount of maltose as a stabilizing agent for transport to Lima, Peru, where the vaccine trial was conducted. Aliquots of GST and MBP, for use as negative controls, were also prepared for the vaccine trial. The antigens were reconstituted in sterile de-ionized water immediately prior to vaccination of pigs.

### 2.2 Pig vaccination

The purified GST and MBP fusions of TSOL16, TSOL45-1A and TSOL45-1B were tested in a pig vaccine trial against challenge infection with *T. solium*. The study was reviewed and approved by the Animal Ethics Committee of the School of Veterinary Medicine, Universidad de San Marcos, Lima, Peru. Twenty 8-week old piglets were obtained from a cysticercosis free farm located in Huaral, Lima. Animals were divided into four groups of 5 pigs each. All animals were vaccinated against Classical Swine Fever prior to the start of the trial. Each pig received 200 μg of antigen and 1 mg Quil A (Brenntag Biosector, Denmark) per immunization in a 1 ml dose. Immunizations were given intramuscularly in the right hind-quarter via a 0.9 mm × 38 mm needle and 1 ml syringe (Becton Dickinson, U.K.). Piglets received their first immunization with recombinant antigen prepared as a GST fusion. Pigs received a second, identical immunization approximately four weeks after the first immunization. Two weeks after the second immunization, pigs were given a third immunization with recombinant proteins prepared as MBP fusions. Pigs in the control group received GST in the first two immunizations and MBP in the third, all in the presence of 1 mg Quil A.

Blood samples were obtained from the jugular vein of all animals at weekly intervals from the first immunization until thirteen weeks later using 10 ml vacutainers (Becton Dickinson, U.K.) and 18 gauge needles. Serum was separated by centrifugation and stored at −20 °C.

### 2.3 Parasites and parasite infections

Pigs were challenged with *T. solium* eggs within a single gravid proglottid as described in [5] two weeks after the third immunization and necropsied approximately 3 months after the last immunization. Four different worms were used for supply of the gravid proglottids. The segments from the four worms were randomly distributed to pigs in the various experimental groups.

Carcass muscle was examined for the presence of cysticerci from the challenge infection by slicing at approximately 3 mm intervals. In carcasses which were heavily infected with cysticerci, the total number in muscle were estimated by selecting a muscle sample (of known weight) from the carcass, determining the number of cysticerci in that sample and estimating the total number in the remaining muscle using its weight.

The Mann–Whitney *U* test was used for comparison of the number of *T. solium* cysticerci found in pigs in different groups immunized with the various antigens. A two-tailed *P* value <0.01 was considered to be statistically significant.

### 2.4 Serological analysis

Specific antibody levels against TSOL16, TSOL45-1A or TSOL45-1B were determined using an enzyme-linked immunosorbent assay (ELISA) as described in [17]. The level of antibody to the specific parasite antigens rather than to the affinity tag (GST) was measured by coating ELISA plates with parasite antigen fused to MBP. Binding of porcine antibody to the MBP fusion proteins of the recombinant antigens was detected using anti porcine IgG-horse radish peroxidase conjugate (Serotec). Antibody titres were calculated from the highest serum dilution at which the optical density at 450 nm equalled 1.0.

Antigenic cross-reactivity was investigated by direct ELISA and inhibition ELISA as detailed by Assana et al. [18]. Briefly, direct ELISA utilized TSOL18-MBP for coating the ELISA wells and application of anti-TSOL16 serum for investigations into antigenic relatedness. The ability of the heterologous recombinant proteins (TSOL18, TSOL45-1A) to inhibit binding of anti-TSOL16 antibodies to homologous antigen (TSOL16) was investigated by antibody inhibition ELISA. Inhibitory antigens were premixed with antibody prior to the addition of the mixture to antigen coated wells.

## 3 Results

### 3.1 Cysticercosis infection

The number of *T. solium* cysticerci detected in each pig is shown in Table 1. Cysticerci were found in each of the 5 control pigs vaccinated with GST and MBP, ranging from 22–3831 cysts per animal (mean = 961). In the group of pigs immunized with TSOL16, two animals contained no cysts, two pigs contained one cyst each and one pig contained six cysts (mean = 2, range = 0–6). Pigs vaccinated with TSOL16 showed a significant reduction in the number of cysticerci compared with those in the control group immunized with GST/MBP (99.8% protection, *P* = 0.008). Pigs belonging to the group immunized with the TSOL45-1A antigen were all found to be infected and contained between 1–63 cysticerci per animal (mean = 20), representing a 97.9% reduction in the mean number of parasites found in control animals (961), however statistical comparison of the group immunized with TSOL45-1A and the controls did not find the groups to be significantly different (*P* = 0.087, Mann–Whitney *U* test). The group of pigs vaccinated with TSOL45-1B contained between 18–2912 cysticerci per animal (mean = 780), showing no statistical difference compared with the control group (*P* > 0.99).

### 3.2 Immune responses

**Fig. 1:**
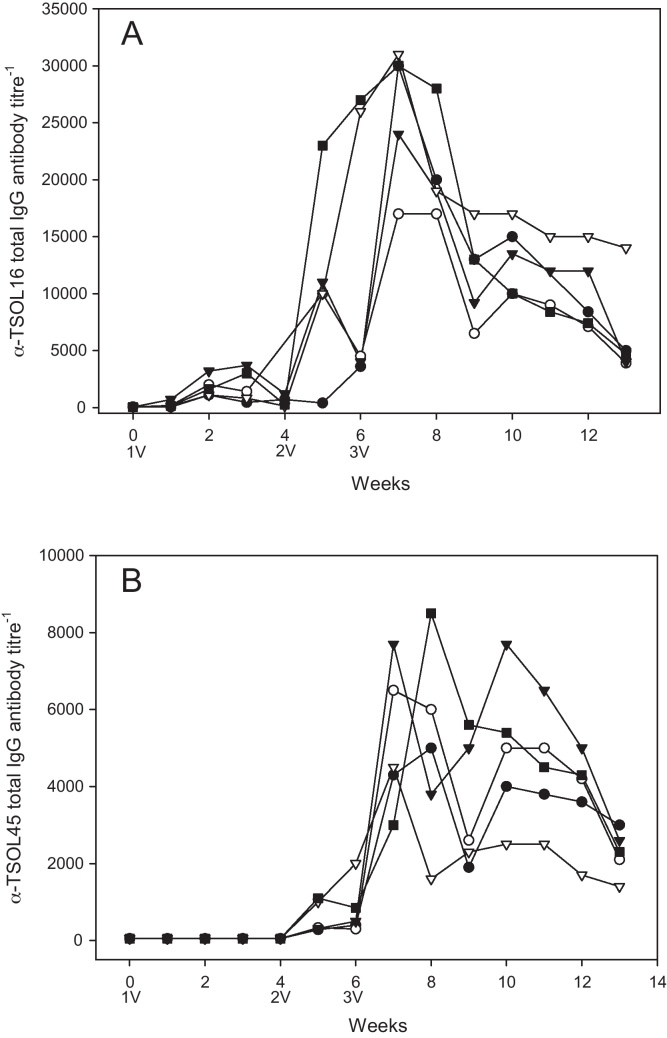
Specific antibody titres, measured by ELISA, in individual pigs immunised with TSOL16 (A) or TSOL45-1A (B). 1V: First immunisation, 2V: second immunisation, 3V: third immunisation. Each curve represents the immune response of a single animal.

Serological analyses of pig sera from samples taken throughout the vaccine study indicate that specific immune responses to the recombinant antigens were produced in the vaccinated animals, with clear rises in total IgG titres observed after the second and third immunizations (Fig. 1). Pigs immunized with TSOL16 produced specific IgG antibodies characterized by increased immune responses following primary and secondary immunization (Fig. 1A). Detectable antibody titres could be measured one week after the first TSOL16 immunization, with peak antibody titres (approximately 17,000–31,000; mean = 26,400) raised in pigs vaccinated with TSOL16 one week following the third immunization. No reactivity was seen with any serum samples in ELISA to MBP, including the sera taken 2 weeks after the immunizations that had involved the use of MBP fusion proteins (i.e. the third immunization).

Pigs vaccinated with TSOL45-1A (Fig. 1B) had measurable antibody titres one week after the second immunization, with peak titres (3000–7700; mean = 5200) occurring 1 week after the third immunization. Control pigs not vaccinated with TSOL16 or TSOL45-1 showed no detectable level of antibody to these proteins throughout the study. Mean peak antibody titre for pigs immunized with TSOL16 (26,400, Fig. 1A) was higher compared with peak antibody titres in pigs vaccinated with TSOL45-1A (5200, Fig. 1B). Pigs immunized with TSOL16 were challenged with *T. solium* eggs when anti TSOL16 antibody titres were estimated as being between 17,000–28,000 (mean = 20,600), while pigs vaccinated with TSOL45-1A were challenged when anti TSOL45-1A antibody titres ranged from 1600–8500 (mean = 5000).

Immunological assessment of pigs vaccinated with TSOL45-1B (two weeks after the second immunization) showed they all had detectible immune responses to TSOL45-1B (antibody titres of 450–2000) and that immune responses in these pigs were generally higher to TSOL45-1B than to TSOL45-1A (50–1700). Immune responses in pigs vaccinated with TSOL45-1A were higher to TSOL45-1A (300–2000) than to TSOL45-1B (50–650).

No clear relationship was apparent between the titre of specific antibody measured to the individual vaccine antigens and the number of cysticerci detected at necropsy following the challenge infection with *T. solium*. Pig antiserum raised against TSOL16-GST showed no cross-reactivity with TSOL18-MBP in direct ELISA. Similarly, pig antisera raised against-TSOL18-GST showed no cross-reactivity with TSOL16-MBP. In inhibition ELISAS, addition of the homologous combinations of antigen and antisera (TSOL16 and anti-TSOL16, TSOL18 and anti-TSOL18) led to total inhibition of the sera's reactivity in ELISA, however no inhibition was evident when heterologous combinations of antigen and antisera (TSOL16 and anti-TSOL18, TSOL18 and anti-TSOL16) were used (data not shown).

## 4 Discussion

The results of the vaccine trial in which pigs were immunized with the TSOL16 recombinant antigen demonstrates that the antigen is able to confer high levels of protection against challenge infection with *T. solium* (Table 1). The homologous antigen from *T. ovis*, To16, was first identified from an oncosphere cDNA library by immuno-screening with antiserum raised against a 16 kDa oncosphere antigen [9], following experimental fractionation of protein extracts of the oncosphere and testing these extracts in sheep vaccine trials. The resulting To16 recombinant antigen was shown to reduce *T. ovis* infection in vaccinated lambs by 92%. These findings provided the basis for identifying a homologous antigen in *T. solium* [8], thereby eliminating the requirement for testing of native *T. solium* antigens in pig vaccine trials and increasing the likelihood of isolating a recombinant antigen that is protective against *T. solium* cysticercosis. A similar strategy was successful for developing the TSA9/TSA18 vaccine for *T. saginata* [19] and the TSOL18 vaccine antigen against porcine cysticercosis [4,20]. The host-parasite relationship in cestodes offers a number of advantages in relation to the likelihood of successful development of vaccines [21], nevertheless the successes that have been achieved with cestode parasites contrasts with broader strategies based on genomic/transcriptomic/proteomic studies [22–27] where isolation of large numbers of candidate vaccine antigens can be problematic for the discovery of protective antigens.

In the experiment described here, TSOL45-1A did not provide statistically significant levels of protection against *T. solium* infection (Table 1). This contrasts, however, with previous studies which demonstrated that pigs vaccinated with TSOL45-1A can be protected against *T. solium* infection [4,5]. Flisser et al. [4] were able to demonstrate protection in pigs vaccinated with TSOL45-1A, with these animals having a higher mean IgG1 titre (approximately 13,000) at challenge compared to total IgG antibody titres to TSOL45-1A in pigs vaccinated with the same antigen in this study (Fig. 1B, mean = 5200).

Variability in the level of infection obtained between individual animals may have affected the capacity of the vaccine trial described here to achieve statistical significance between some of the different treatment groups. In the study undertaken by Flisser et al. [4] pigs were given eggs isolated from gravid *T. solium* segments such that individual animals received directly comparable challenge infections. In the trial of TSOL45-1A where statistically significant protection was achieved [4] the twelve control animals harboured between 6 and 127 cysts, representing a range varying by a factor of 21 from lowest to highest. In Peru where the trial described here was undertaken, greatest success has been achieved in experimental infections in pigs by giving whole gravid proglottids rather than isolated eggs, however a disadvantage of the method is the necessity to use different adult worms to supply the proglottids and individual animals also receiving different proglottids [28]. In the experiment described here, this led to a variation in the levels of infection in controls by a factor of 174 between the lowest and highest values (22–3831 cysts). In this case, it is difficult to interpret whether the TSOL45-1A vaccinated animals that had 25 and 63 cysts were either non-protected or >98% protected depending on whether they received the lower or higher infective dose delivered to the control animals. Nevertheless TSOL16 appeared to be a more effective immunogen than TSOL45-1A in this experiment, with TSOL16-vaccinated animals being both statistically significantly protected in comparison to controls as well as having statistically significant fewer cysts than the TSOL45-1A vaccinates (*P* < 0.05).

The oncosphere antigens of cestode parasites are typically problematic to express in *E. coli* [19,29,30] and GST or MBP fusion proteins have been used as immunogens because these have advantages in regard to expression level and solubility compared to the non-fused or HIS-tagged antigens. Here we used a vaccination strategy incorporating both GST and MBP fusion proteins of the same antigen in an attempt to boost immune responses to the parasite-derived portion of the recombinant antigens. The first two immunizations given to the pigs each contained the oncosphere antigens fused to GST. The third immunizations each contained the antigens fused to MBP, the aim being to boost immune responses to the parasite-encoded portions of TSOL16, TSOL45-1A or TSOL45-1B rather than to the GST fusion partner. Previous studies have shown that a substantial portion of the antibody response in pigs [17] and sheep [31,32] is raised against the highly immunogenic GST fusion partner. Responses to both TSOL16 and TSOL45-1A were substantially greater after the third immunization compared with responses after the second (Fig. 1). This suggests that the strategy of utilizing different fusion partners for the immunization may have enhanced responses to the parasite-encoded component of the immunogen. However no animals received three immunizations using GST only and hence a clear interpretation cannot be made about the advantage of using different fusion protein partners to enhance vaccine responses. Comparisons between the immunogenicity of TSOL45-1A and TSOL45-1B were inconclusive since statistically significant levels of protection were not achieved with either antigen in this study. Had protection of pigs with TSOL45-1A (containing two FnIII domains) been demonstrated, as in the two previous studies [4,5], comparisons between TSOL45-1B (one FnIII domain) and TSOL45-1A may have provided further information about the position of host protective epitopes within the latter antigen. By comparison, the TSOL16 and TSOL18 antigens each consist of a single FnIII domain and both have now been shown to protect pigs against *T. solium* infection. Linear B-cell epitopes within the FnIII domain of TSOL18 have been identified [17], although current data suggests that the dominant antibody specificities to TSOL18 from immunized pigs appear to be directed toward conformational epitopes [18].

TSOL16 appears to be specifically expressed in the larval oncosphere stage of the parasite that infects pigs [10] and is associated with the penetration gland cells within *T. solium* [11]. Future studies may focus on more detailed investigations to elucidate the function of TSOL16 in the oncosphere during infection of pigs and identification of the host protective epitopes within the antigen.

The results achieved in this study indicate that the TSOL16 antigen could be a valuable adjunct to porcine vaccination with TSOL18 and may allow the further development of new vaccination strategies against *T. solium* cysticercosis.

## Figures and Tables

**Table 1:** Number of *T. solium* cysticerci in pigs immunized with recombinant antigens and challenged with *T. solium*.

Group (antigen)	Number of cysts in individual pigs	Mean	*P* value^a^	Protection^b^ (%)
Control	22, 31, 34, 889, 3831	961	–	–
TSOL16	0, 0, 1, 1, 6	2	0.008	99.8
TSOL45-1A	1, 5, 5, 25, 63	20	0.087	97.9
TSOL45-1B	18, 93, 127, 750, 2912	780	>0.99	18.8

a The Mann–Whitney *U* test was used for comparison of the number of cysticerci in vaccinated pigs compared with controls.

b Calculated as the percentage reduction in the mean number of cysticerci in comparison with controls.
